# Alzheimer’s Disease-Related Genes Identified by Linking Spatial Patterns of Pathology and Gene Expression

**DOI:** 10.3389/fnins.2022.908650

**Published:** 2022-06-14

**Authors:** Roger Mullins, Dimitrios Kapogiannis

**Affiliations:** Laboratory of Neurosciences, National Institute on Aging, Baltimore, MD, United States

**Keywords:** tau, FDG-18, PLS-DA, ADNI, allen human brain atlas, Alzheimer’s disease, Alzheimer’s, amyloid-β

## Abstract

**Background:**

Alzheimer’s Disease (AD) is an age-related neurodegenerative disease with a poorly understood etiology, shown to be partly genetic. Glucose hypometabolism, extracellular Amyloid-beta (Aβ) deposition, and intracellular Tau deposition are cardinal features of AD and display characteristic spatial patterns in the brain. We hypothesize that regional differences in underlying gene expression confer either resistance or susceptibility to AD pathogenic processes and are associated with these spatial patterns. Data-driven methods for the identification of genes involved in AD pathogenesis complement hypothesis-driven approaches that reflect current theories about the disease. Here we present a data driven method for the identification of genes involved in AD pathogenesis based on comparing spatial patterns of normal gene expression to Positron Emission Tomography (PET) images of glucose hypometabolism, Aβ deposition, and Tau deposition.

**Methods:**

We performed correlations between the cerebral cortex microarray samples from the six cognitively normal (CN) post-mortem Allen Human Brain Atlas (AHBA) specimens and PET FDG-18, AV-45, and AV-1451 tracer images from AD and CN participants in the Alzheimer’s Disease and Neuroimaging Initiative (ADNI) database. Correlation coefficients for each gene by each ADNI subject were then entered into a partial least squares discriminant analysis (PLS-DA) to determine sets that best classified the AD and CN groups. Pathway analysis *via* BioPlanet 2019 was then used to infer the function of implicated genes.

**Results:**

We identified distinct sets of genes strongly associated with each PET modality. Pathway analyses implicated novel genes involved in mitochondrial function, and Notch signaling, as well as genes previously associated with AD.

**Conclusion:**

Using an unbiased approach, we derived sets of genes with expression patterns spatially associated with FDG hypometabolism, Aβ deposition, and Tau deposition in AD. This methodology may complement population-based approaches for identifying the genetic underpinnings of AD.

## Introduction

Alzheimer’s disease (AD) is a progressive neurodegenerative disease that accounts for 60–70% of dementia cases in the aging population. The pathophysiology of the disease includes glucose hypometabolism, whereas its cardinal neuropathological features are the accumulation of aggregates of amyloid beta-peptide (Aβ) in extracellular plaques and intracellular hyperphosphorylated tau tangles. Pathologic forms of these proteins and their aggregates impair synaptic function and induce maladaptive neuroinflammation involving astrocytes and microglia. This process eventually results in synaptic and neuronal loss, macroscopically evident as brain atrophy (De Strooper and Karran, 2016; Frisoni et al., 2022). Although the proximate causes for Aβ and Tau aggregation have been largely crystalized in the “amyloid hypothesis,” the ultimate causes of AD remain unknown (Frisoni et al., 2022). Specific brain regions, such as the medial temporal, precuneus/posterior cingulate, lateral temporoparietal cortices, are more prone to develop severe AD pathologies and manifest them earlier during the disease. By contrast, other regions such as the primary motor cortex, sensory cortex and cerebellum remain almost intact (Frisoni et al., 2022). Attempts to explain this selective regional vulnerability have focused on the structural and functional connectivity of the default mode network (Buckner et al., 2009; Seeley et al., 2009) and the spatial interplay of distinct processes leading to glucose hypometabolism, Aβ plaques, and Tau deposition within networks (Sepulcre et al., 2016).

The pathogenic cascade of AD extends over decades and follows a characteristic regional progression, starting in distinct brain regions for Aβ and Tau (Arnold et al., 1991; Braak and Braak, 1991; Braak and Del Tredici, 2012; Sepulcre et al., 2016). AD pathology is preceded or accompanied by changes in the expression of many genes. The brains of late-stage AD patients exhibit severe neuronal loss, which could result in an altered gene expression profile. The underlying spatial patterns of gene expression have been shown to account for both structural (Burt et al., 2018; Reardon et al., 2018) and functional (Richiardi et al., 2015; Vertes et al., 2016) features in the human brain, and similar methods have been used successfully to examine genes implicated in disease states such as Parkinson’s (Keo et al., 2021) and Huntington’s disease (McColgan et al., 2018).

Given that the distribution of most gene expression varies widely throughout the brain, we previously hypothesized that regional differences in normal gene expression during young to middle age may relate to or mediate regional vulnerability to Aβ and Tau pathologies (Diehl et al., 2017; Mullins et al., 2017). In prior studies, we focused on limited sets of genes associated with insulin resistance, and revealed compelling associations between the Brodmann area topography of normal expression of metabolism and insulin signaling-related genes, and those of established (Arnold et al., 1991) pathological Aβ and Tau.

**FIGURE 1:**
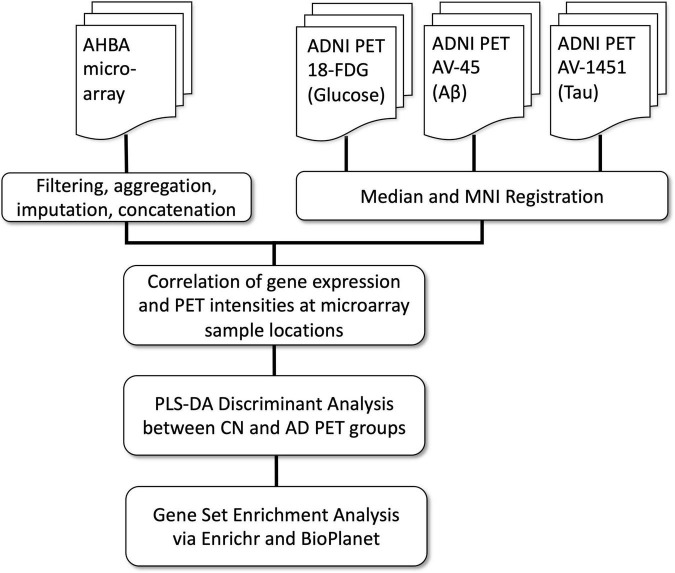
Processing and analysis flow chart.

In the present study we expand this hypothesis to investigate whether normal regional cortical differences in gene expression are related to the cardinal pathological features of AD, and to use this information to identify specific genes and pathways related to AD pathology. Given the striking and well-characterized regional differences in glucose hypometabolism, Aβ and Tau, we focused on these intermediate disease phenotypes. To establish reliable image maps of these pathologies, we used FDG-18 (glucose metabolism), AV-45 (Aβ), and AV-1451 (Tau) PET scans from the large ADNI cohort of AD and CN subjects. Next, we examined the spatial correlation of these maps with co-registered maps of gene expression from the Allen Human Brain Atlas (AHBA)(Hawrylycz et al., 2012). We then used the resulting correlation coefficients, one for each gene per subject, as inputs to a Partial Least Squares Discriminant Analysis (PLS-DA). Underlying this approach is PLS regression (PLS-R) (Wold et al., 2001), a flexible Principal Components Analysis-based method often used to assess commonalities between AHBA transcriptome data and 3D imaging data from other modalities. Specifically, PLS-R has been used to find the spatial correlation between AHBA gene expression and resting-state functional connectivity in healthy subjects (Vertes et al., 2016; Zhu et al., 2021), and with regional cortical thickness changes in Parkinson’s (Keo et al., 2021) and Huntington’s disease (McColgan et al., 2018). An assumption of PLS-R is that the system under investigation is primarily influenced by a small set of underlying “latent” variables which are maximally correlated between the datasets. PLS-DA extends this method toward classification, regressing binary group variables against a corresponding set of predictor variables (Perez-Enciso and Tenenhaus, 2003). See Figure 1 for a flowchart of this process.

It is worth noting that this method does not intend to reveal the actual spatial distribution of gene expression in the disease condition, only that a pathology is more or less correlated spatially with a given gene expression pattern. The rationale for conducting a correlative analysis between data obtained from individuals at different age groups is provided by the natural history of AD: AD pathologies start developing in young-middle age in brain areas with different transcriptomic signatures, these pathologies evolve over time in varying degrees for different brain areas and culminate at distinct patterns of pathology in older brains. Given that gene expression was assessed in the brains of individuals who died young or in mid-life, before the typical age when AD pathologies begin accumulating, the correlations may reveal genes implicated in the mechanisms conferring regional resilience or vulnerability to the development of AD.

This study demonstrates a novel data-driven bioinformatic approach using the spatial correlation between normal gene expression and image intensity of three types of PET conducted in AD and Cognitively Normal (CN) individuals as input to a discriminant analysis. Our specific hypothesis is that the spatial patterns of emergent pathologies in the AD brain are associated with the normal spatial expression of specific genes. Our primary aim was to use this method to derive sets of genes for optimal classification of AD and CN individuals based on their PET measures of FDG-18 hypometabolism, Aβ, and Tau deposits. As a secondary aim, we sought to identify novel genes associated with distinct aspects of AD pathology and uncover biological processes that may contribute to their development.

## Materials and Methods

### Participants

#### Alzheimer’s Disease and Neuroimaging Initiative Participants

Baseline FDG-PET, AV45-PET (Aβ), and AV1451-PET (Tau) images from the ADNI Image & Data Archive site^1^ were downloaded as Neuroimaging Informatics Technology Initiative (NIFTI) file format volumes in January of 2022. We analyzed each PET tracer for CN and AD ADNI participants ranging from 55 to 95 years old (Table 1). ADNI was launched in 2003 by the National Institute on Aging (NIA), the National Institute of Biomedical Imaging and Bioengineering (NIBIB), the Food and Drug Administration (FDA), private pharmaceutical companies and non-profit organizations, as a $60 million, 5-year public-private partnership. The primary goal of ADNI has been to test whether serial magnetic resonance imaging (MRI), PET, other biological markers, and clinical and neuropsychological assessment can be combined to measure the progression of MCI and early AD. The Principal Investigator of this initiative is Michael W. Weiner, MD, VA Medical Center and University of California San Francisco. For up-to-date information, see www.adni-info.org.

**TABLE 1:** Positron emission tomography (PET) pathology image demographics.

PET modality	Group	N	Age (yrs ± SD)	Age (range)	Sex (F:M)
FDG-18	AD	305	75.33 ± 7.41	55–91	123:182
	CN	351	75.26 ± 5.93	56–94	177:174
AV-45	AD	174	74.59 ± 8.42	56–90	73:101
	CN	421	74.27 ± 7.30	56–95	228:193
AV-1451	AD	65	74.35 ± 8.47	56–89	24:41
	CN	435	73.13 ± 7.90	55–94	248:187

#### Allen Human Brain Atlas Human Brain Specimens

The Allen Human Brain Atlas (AHBA) incorporates microarray data from six postmortem brain specimens obtained from normal donors with no known prior neuropathological or neuropsychiatric history (Hawrylycz et al., 2012). Each specimen provided 501–946 distributed sample sites for the microarray set of 29,191 unique genes, with multiple probes available for 93% of these genes. Detailed donor profile information is available in Supplementary Table 1 and the http://human.brain-map.org/ documentation section.

### Allen Human Brain Atlas Microarray Data Preprocessing

Data for the probes, sample sites, and normalized expression values was imported from the files available for download at the Allen Institute for Brain Science, Allen Human Brain Atlas site: http://human.brain-map.org/static/download. Detailed information and white papers for the survey, platform selection, and normalization of the Agilent 8x60K custom microarray data is available at the http://human.brain-map.org/ documentation section. These consisted of 58,692 probes (replicates for the 29,191 genes) for each sample. Preprocessing was performed using R (v.4.1.0) and the Bioconductor package (Biobase v.2.5.2). The following steps were applied: (1) Removed AHBA microarray probes with no gene ontology (GO) annotation or entrez-id, leaving 43,714 probes. (2) Set sample values with expression values below background as missing “NA” *via* the present-absent call (PAC) files provided in the AHBA data, then removed probes missing more than 50% of the samples within any specimen, leaving 27,349 probes. (3) To further reduce missing values, improve signal, and enable gene set expression analysis, we selected the “best” probe for each gene using the WGCNA library *collapseRows* function and the “*MaxMean*” method. This selected the row with the highest mean value within a probe or the highest connectivity among the rows if three or more probes were available. This aggregation reduced the number of probes to the final 13,753 individual genes used in the rest of the analysis, with only 3.4% of the values missing. (4) Missing value imputation was performed on the microarray data for each of the six specimens individually *via* the missMDA (v. 1.18) package *imputePCA* function, which uses a principal components analysis to impute missing values (Josse and Husson, 2016). The microarray data for each of the six donors was then concatenated into one profile. Only the 2,754 samples from the cerebral cortex were included in this analysis, as the cerebellum and brain stem are largely spared by AD and could drive spurious associations due only to systematic genome-wide differences in expression levels between these regions (Kang et al., 2011; Mahfouz et al., 2015).

### Alzheimer’s Disease and Neuroimaging Initiative Positron Emission Tomography Image Processing

**FIGURE 2:**
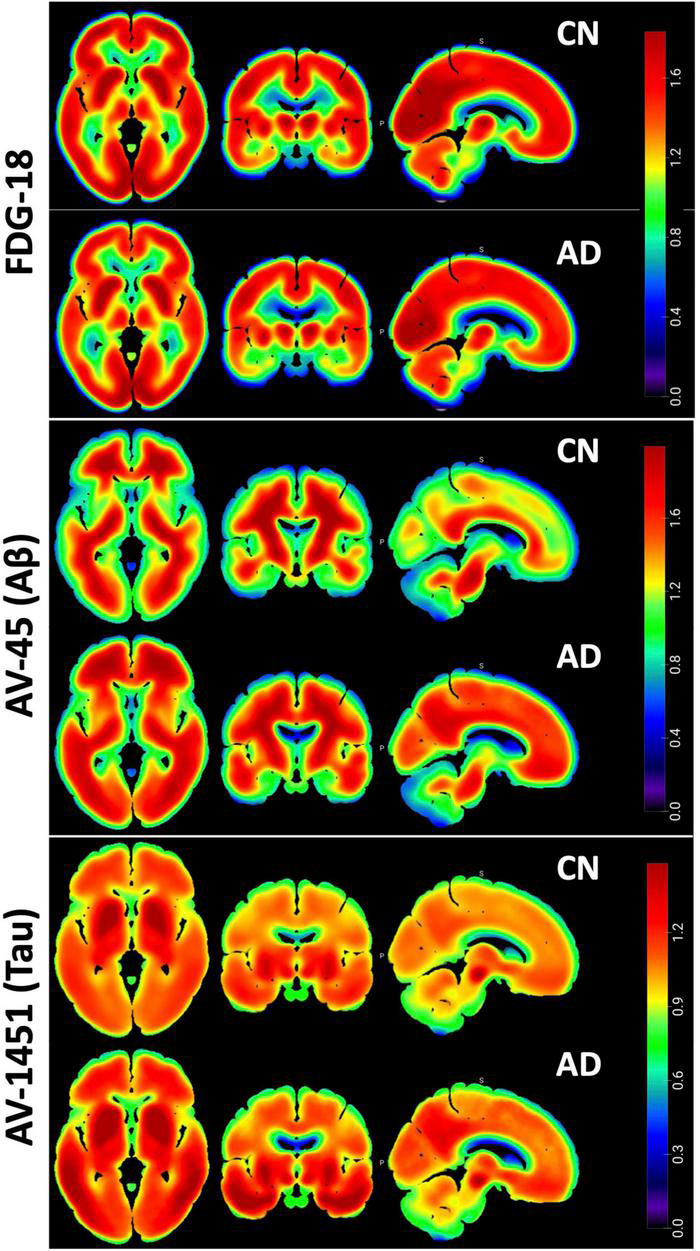
Pet Pathology Z-maps. Multi-planar Axial, Coronal, and Sagittal views of the median of the MNI-registered ADNI AD and CN participant images. Top row is FDG-18 maps of hypometabolism, middle is AV-45 Aβ distribution, bottom is AV-1451 Tau distribution. FDG-18 images were normalized to the median of a manually drawn pons ROI. AV-45 and AV1451 were normalized to whole cerebellum. Thresholding for the figures is consistent between AD and CN groups for visual comparison. Non-brain areas are masked and the “NIH” look-up-table was used for color scale. Figure created in MRIcroGL v1.2.

All PET images were fully preprocessed by ADNI, including smoothing, coregistration, frame averaging, AC-PC orientation, and intensity normalization. Each individual pre-processed image was registered to the median image for that modality *via* FSL-flirt, which was then registered to the T1 152-subject MNI (Montreal Neurological Institute) standard template and manually inspected for accuracy of registration. All images were then co-registered to the MNI template using that transform. See Figure 2 for aggregate images in each modality.

### Spatial Correlation

The first step in this process was to obtain matching PET intensity values for the brain locations sampled by the AHBA microarray. A custom R script using RNifti (version 1.4.0) was used to load in each PET image and read the intensity values at the coordinates specified in the AHBA data. An optimized set of ANTs (Avants et al., 2011) nonlinear-registered MNI coordinates^2^ was used as corresponding microarray sample locations for the AHBA and PET images. This provided tabular output with each row being an ADNI participant and each column the PET intensity in each of the 2,754 cerebral cortex sample locations from the six AHBA specimens. This was repeated to create a separate data table for each PET modality.

Next, correlation coefficients as *r*-values were derived as pairwise distances *via* the dist2 function in MATLAB (Mathworks, Natick, MA, United States), using a Pearson correlation metric. This correlation was performed between the single concatenated set of AHBA gene expression values and the PET pathology intensities for each ADNI participant in the same AHBA sample locations. This correlation was repeated within each PET modality, resulting in a new data table with each row being an ADNI participant and each column being the respective *r*-values for each gene. These *r*-values were then converted to z-scores using the Fisher r-to-z transform and entered into the following PLS-DA model.

### (Sparse)PLS-DA

Starting with a table of *z*-values reflecting the spatial correlation between gene expression and PET pathology in the cerebral cortex microarray samples, we used the sparse PLS-DA (sPLS-DA) from the mixomics (Rohart et al., 2017) R package (v.6.16.3) to perform a discriminant analysis between Alzheimer’s disease (AD) and cognitively normal (CN) ADNI participants. Sparse PLS-DA classified the samples based on the best predictive or discriminative features in a one-step procedure (Le Cao et al., 2011). The table of *z*-scores was used as the input dataset and the AD or CN diagnosis as the classifier. The model was tuned using the *tune.splsda* function with leave-one-out (loo) validation and 50 repeats. The tuning function consistently revealed that the optimal number of components was two for each PET pathology. The optimal number of classification variables for components 1 and 2, respectively were 6 & 10 for FDG, 30 & 20 for AV45, and 35 & 5 for AV1451.

### Gene Set Enrichment Analysis

Enrichr (Chen et al., 2013; Kuleshov et al., 2016; Xie et al., 2021) was used to query the gene sets derived from the PLS-DA analysis above, using the *enrichR* version 3.0 package in R. The gene sets derived from the first principal component of the PLS-DA step were used as inputs for the enrichment analysis individually and assessed with BioPlanet 2019 (Huang et al., 2019), which integrates pathways from curated sources including the Kyoto Encyclopedia of Genes and Genomes (KEGG), NCI-Nature, BioCarta, Science Signaling, Reactome, NetPath, and WikiPathways.

## Results

### Sparse PLS-DA Gene Selection

**FIGURE 3:**
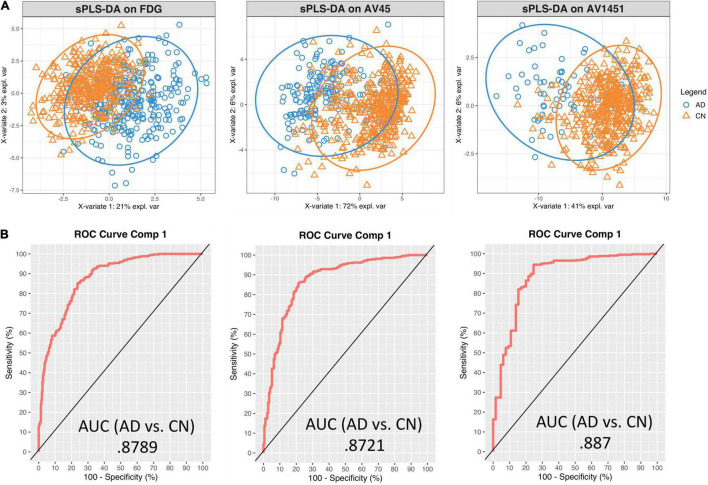
Sparse PLS-DA plots for gene selection. **(A)** Sample plots from sPLS-DA performed on the gene expression – PET intensity correlation data including 95% confidence ellipses. The samples are projected into the space spanned by the first two components and colored by group: blue for AD and orange for CN. **(B)** Receiver Operator Characteristic (ROC) curve and AUC on the expression-intensity correlation data for component 1.

Sparse PLS-DA was used to identify the optimal set of genes whose expression-intensity correlation value discriminated between CN and AD participants. Separate models were created for FDG-18, AV-45, and AV-1451 PET, as described above. The first component of the PLS-DA analysis for FDG-18 explained 21.2% of the variance, AV-45 71.6%, AV-1451 41.4%. The first component was retained for further analyses, as the variance explained by the second components for FDG-18, Aβ, and Tau distribution was marginal: 2.3, 2.6, and 2.6%, respectively. Sample plots for each PET pathology showing the distribution of the data in latent space are in Figure 3A.

Receiver Operator Characteristic (ROC) Area Under the Curve (AUC) plots were used to further evaluate the classification results. Results were similar between each pathology. For discrimination between AD and CN on the first component, the FDG-18-associated set had an AUC of 0.88, the AV-45-associated set an AUC of 0.87, and the AV-1451-associated set an AUC of 0.89. AUC curves presented in Figure 3B are for comparison only, as they are generated using specificity and sensitivity cutoff maximization rather than PLS-DA distance metrics (Rohart et al., 2017).

**FIGURE 4:**
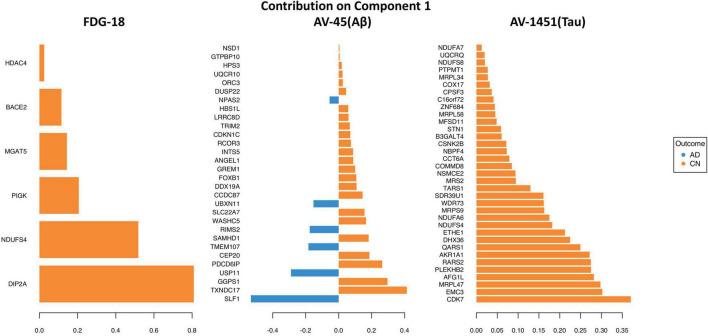
Loading plots for the optimal classifying genes in the sPLS-DA analysis. Highest loading genes or pathologies are on the bottom (descending order), leftward (blue) deflected bars classify to AD, rightward (orange) to CN.

Genes selected by the sPLS-DA model are shown in the loading plots, which show the direction each expression-intensity correlation classifies toward (Figure 4). For genes that classified toward AD, their underlying average expression-intensity correlation was higher in the AD group. Likewise, genes that classified toward CN had higher expression-intensity correlations in the CN group. This signifies that the spatial pattern of the PET image intensity diverged far enough in either direction from the spatial pattern of normal gene expression that it would aid in classification. See Supplementary Tables 2, 3 for annotations and loading statistics output for these genes.

### Gene Set Enrichment Analysis

**FIGURE 5:**
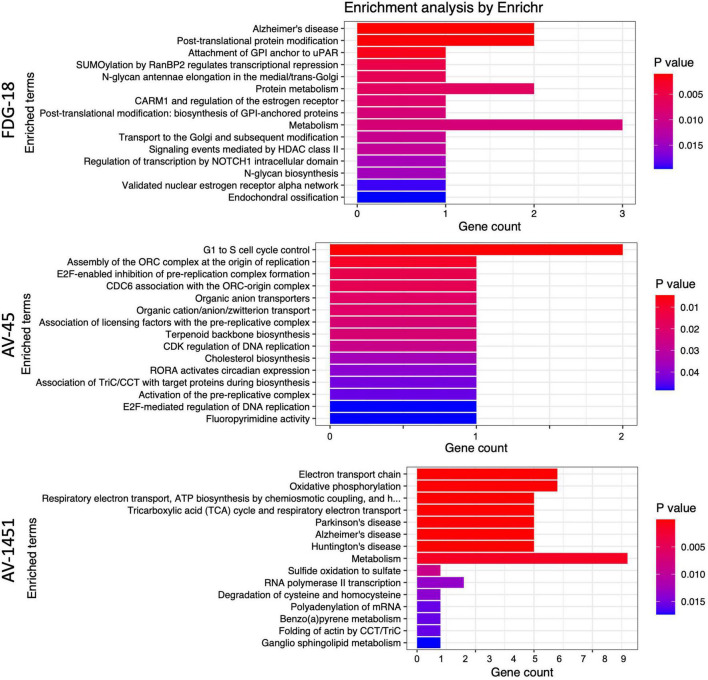
Enrichr results as barplots for FDG-18, AV-45, and AV-1451 PET gene sets. These figures are limited to the top 15 significant pathways (*p* < 0.05), the complete set is in Supplementary Table 3. The *x*-axis categories are the BioPlanet pathways arranged by *p*-value from lowest (top) to highest (bottom). The *y*-axis is the gene count, or number of genes found in that pathway.

Enrichr was used to examine the biological relevance of groups of genes within the selected gene sets according to the BioPlanet 2019 pathway set. Plots of the top 15 significant (*p* < 0.05) pathways for each pathology by *p*-value and gene count are shown in Figure 5. The overlap with many Bioplanet pathway gene sets is unavoidably low due to the optimal small size (6–35 genes) of the sPLS-DA derived classifier sets, so this analysis is an exploratory measure to infer function. Correcting for multiple comparisons by the Benjamini-Hochberg (BH) procedure, the adjusted *p*-values for FDG-18 retained all pathways, those of AV-1451 exceeded a p of.05 after the 8th listed pathway (Metabolism), and AV-45 retained no significant pathways. See Supplementary Table 4 for a full list of pathway outputs, associated genes, and statistics.

## Discussion

Using an unbiased approach, we derived sets of genes with expression patterns spatially associated with FDG hypometabolism, Aβ deposition, and Tau deposition in AD. Pathway analysis of these gene sets *via* BioPlanet revealed links to mitochondrial function, Notch signaling, and other neuropathologically interesting pathways that may underlie the canonically distinct spatial patterns of FDG hypometabolism, Aβ and Tau deposition in AD.

From a broad perspective, the regional patterns of different AD pathologies implicated different sets of genes, with the exception of NDUSF4, which was implicated in regional vulnerability to both Tau and FDG hypometabolism. All sets classified between AD and CN with similar accuracy, with Aβ marginally on the low end and Tau on the highest. FDG reached optimal classification using only six genes, compared to 30 for Aβ and 35 for Tau. All revealed significant and meaningful pathway results, but only FDG and Tau survived correction for multiple comparisons. FDG and Tau also showed higher numbers of genes classifying toward CN in the discriminant analysis, which may imply regional protective effects of these genes against the development of FDG hypometabolism and Tau deposition. Such protective effects are less pronounced for Aβ, which has genes classifying toward either group. Tau and FDG are also the only gene sets that map to a pathway indicative of AD itself, which does not emerge for the Aβ-associated gene set. It is worth noting that these results reflect the current focus of AD research, which is shifting away from the amyloid hypothesis (Morris et al., 2014) and toward Tau (Josse and Husson, 2016) and brain metabolism (Neth and Craft, 2017) as primary pathogenic events of interest.

The nominal “Alzheimer’s disease” pathway is the foremost one identified by BioPlanet for the discriminant gene set for FDG-18, implicated *via* the influence of BACE2 and NDUFS4. BACE2 is the focus of considerable interest in AD as a conditional β-secretase that normally suppresses the amyloidogenic processing of APP (Huentelman et al., 2019; Wang et al., 2019). NDUFS4 codes for a mitochondrial subunit known to bind oligomeric Aβ (Olah et al., 2011) and may have a role in the cognitive deficits of AD *via* oxidative stress (Harris et al., 2007). The full BioPlanet pathway list in Supplementary Table 4 also revealed numerous several entries for NOTCH signaling *via* the influence of HDAC4, a histone deacetylase with an important role in nerve function by promoting neuronal apoptosis (Bolger and Yao, 2005) and of interest as a therapeutic target for AD due to its deregulation and accumulation in the AD brain (Xu et al., 2011; Shen et al., 2016; Wu et al., 2016). Recent evidence has suggested that aberrant Notch signaling could result in the neurodegeneration seen in AD (Woo et al., 2009; Kapoor and Nation, 2021). In addition, the failure of γ-secretase inhibitors as treatments of AD has been partly attributed to its deleterious effects on Notch signaling, which may have counteracted any benefits from reduced Aβ production (Luo and Li, 2022). MGAT5 was implicated as part of Golgi metabolic pathways and has attracted recent interest due to its human-specific differential expression in brain tissue layers as well as in AD (Jorge et al., 2021). PIGK, and DIP2A were also high classifier loadings in the PLS-DA. Potentially a novel candidate gene, PIGK has little current implication in AD literature but is linked to the maturation or modification of APP (Del Prete et al., 2017). Similarly, the function of DIP2A is still unclear, but it was the strongest loading gene in our FDG-18-related analysis and has been associated with amyloid burden in epigenome-wide association (EWAS) studies of AD using post-mortem brain tissue (De Jager et al., 2014; Li et al., 2020).

Of the three AD pathologies probed by PET imaging, Tau deposition (by AV-1451) appears to reveal the most relevant pathways related to AD, as well as including “Alzheimer’s disease” itself as the most highly significant BioPlanet-identified pathway *via* NDUFS4 & 8, NDUFA6 & 7 and UQCRQ. This “Alzheimer’s disease” pathway overlaps with other AV-1451-associated pathways that relate to mitochondrial respiration, electron transport, and oxidative phosphorylation (NDUFS4 & 8, NDUFA6 &7, UQCRQ, & COX17), as well as metabolism (NDUFS4 & 8, NDUFA6 &7, UQCRQ, COX17, & ETHE1). The mitochondrial subunit NDUSF4 was also found in the FDG-18 gene set as above, through which it shares common features in terms of electron transport, oxidative phosphorylation, and metabolism. Disruptions in these pathways may contribute to both AD metabolic abnormalities and Tau pathology by impairment of mitochondrial function (Yao et al., 2009; Chakravorty et al., 2019; Lim et al., 2020).

In this setting, it is important to note that FDG hypometabolism has long been considered more closely spatially, temporally and causally linked to Tau deposition than Aβ deposition (Ossenkoppele et al., 2016). A common genetic underpinning of mitochondrial and metabolic abnormalities could help account for this relationship. Recently, we identified decreased levels and activity of mitochondrial electron transport chain components in plasma neuronal-derived Extracellular Vesicles of individuals with AD compared to Controls (Yao et al., 2021), as well as in individuals with major depressive disorder (Goetzl et al., 2021) or neuropsychiatric symptoms due to long COVID-19 compared to controls (Peluso et al., 2022). These studies indicate that mitochondrial dysfunction in AD can be studied in living individuals through biomarkers, opening the way to establishing it as a core feature of AD progression.

Many individual genes within the AV-1451 set have been implicated in AD pathogenesis in the past, supporting the validity of our approach. The strongest loading individual gene on the AV-1451 list was the cyclin-dependent kinase CDK7, which is elevated early in AD pathogenesis and may upregulate Amyloid(β) Precursor Protein (APP) and Tau (Zhu et al., 2000; Lukasik et al., 2021). EMC3 is involved in endoplasmic reticulum associated degradation, which has been implicated in neurodegeneration in a mouse AD model (Zhu et al., 2017). AKR1A1 codes for an aldehyde reductase, which is protective against neurodegeneration in AD (Picklo et al., 2001). Differentially methylated positions on B3GALT4 are linked to late onset AD and have been associated with memory performance and CSF levels of Aβ and tau (Madrid et al., 2018). CPSF3 is involved in the RNA life cycle and has been identified as part of the molecular interaction network for AD (Rosenthal et al., 2022). COX17 codes for a cytochrome C oxidase copper chaperone involved in copper homeostastis, which has been tentatively linked to AD (Ejaz et al., 2020). PTMPT1 is part of an AD-risk locus identified *via* genome-wide analyses (Efthymiou and Goate, 2017). However, STN1, AFG1L, PLEKHB2, DHX36, WDR73, SDR39U1, MRS2, NSCME2, COMMD8, CCT6A, NBPF4, SCNK2B, MFSD11, SNF684, and C16orf72, are relatively unstudied in the context of neurodegenerative diseases and AD, raising the possibility of having identified novel mechanisms.

Amyloid-β deposition *via* AV-45 PET revealed the fewest interpretable pathways and did not reveal a significant pathway for “Alzheimer’s disease” (*p* = 0.22). Of the pathways identified, there were some related to the cell cycle *via* CDKN1C and ORC3. Concerning this, there are existing hypotheses that disruptions to cell cycling may be a cause for the neuronal death observed in AD (Raina et al., 1999, 2004) but little in the way of experimental research to test it or the possible role of CDKN1C and ORC3. In terms of individual genes, there were many hints about their involvement in AD pathophysiology. TXNDC17 interacts with the cellular prion protein (PrPc)(Ulbrich et al., 2018), which is the main receptor for oligomeric Aβ. GPPS1 is elevated in the AD frontal cortex and may modify Aβ production (Hooff et al., 2010). PDCD6IP (as ALIX) is decreased in the serum of AD patients and AβPP/PS1 mice (Sun et al., 2015) and directs the trafficking of APP into extracellular vesicles (Cone et al., 2020). RCOR3 is down-regulated in the hippocampus of AD brain specimens (Yan et al., 2019). The tripartite motif protein TRIM2 has high hippocampal expression that may be impacted by the presence of Aβ plaques *via* modulatory miRNA (Schonrock et al., 2012). LRRC8D may interact with Aβ as a binding protein (Virok et al., 2011). DUSP22 inhibits protein-kinase A activity and hence Tau phosphorylation and CREB signaling (Sanchez-Mut et al., 2014). SLF1, UPS11, CEP20, TMEM107, SAMHD1, RIMS2, WASHC5, SLC22A7, UBXN11, CCDC87, DDX19A, FOXB1, GREM1, ANGEL1, INTS5, TRIM2, HBS1NL, NPAS2, UQCR10, HPS3, GTPBP10, and NSD1 are relatively unstudied in this context.

While the spatial correlation was meant to identify genes implicated in the regional vulnerability to AD and not necessarily to improve AD group classification, we also performed a *post-hoc* comparison using only the mean PET intensity for each of the three modalities as the dependent variable, entering each into otherwise identical sPLS-DA models. This resulted in notably lower AUCs: 0.63 for FDG, 0.63 for AV-45, and 0.71 for AV-1451.

A limitation of this study is the fact that while the AHBA contains numerous samples, they are derived from only six brain specimens and from a younger cohort than the ADNI group. The method of spatial correlation we implemented in this study is currently unable to apply covariates for factors such as age and sex, since the spatial correlation involves data derived from two separate sets of subjects. Fortunately, the ADNI participants are consistent in terms of age and sex, and the AHBA specimens have undergone substantial normalization for array and batch-specific biases. Both sex and age interact with gene expression in the brain, particularly in terms of immune activation and metabolism (Berchtold et al., 2008). Until there are comprehensive richly sampled post-mortem studies of regional gene expression with a variety of ages, sexes, and disease statuses, it will be difficult to predict or account for the effects of these potential confounds.

Regarding AD-related genes revealed by genome and epigenome – wide association studies that essentially create binary contrasts of diseased/non-diseased individuals in large populations, we should note that a gene product may still be important in AD without being spatially correlated with a pathology, and vice-versa. The methodology employed in the present study may complement these population-based approaches for identifying the genetic underpinnings of AD. We hope that data-driven methods like ours can identifying novel genes implicated in vulnerability to AD for further evaluation.

## Conclusion

We present a novel method to extract information from the melding of microarray and imaging data to identify genes involved in AD pathology and its regional distribution. This method allowed us to identify both known and novel candidate genes and highlights certain pathways for further investigation, but also as potential therapeutic targets. This methodology is flexible, produces an interpretable list of only the best-classifying genes, and can be extended to provide insight into the genetic underpinnings of other brain diseases with their own characteristic spatial patterns of pathology.

## Author’s Note

Data used in preparation of this article were obtained from the Alzheimer’s disease Neuroimaging Initiative (ADNI) database (https://adni.loni.usc.edu). As such, the investigators within the ADNI contributed to the design and implementation of ADNI and/or provided data but did not participate in analysis or writing of this report. A complete listing of ADNI investigators can be found at: http://adni.loni.usc.edu/wp-content/uploads/how_to_apply/ADNI_Acknowledgments_List.pdf.

## Data Availability Statement

The datasets presented in this study can be found in online repositories. The ADNI PET images used here are available at https://adni.loni.usc.edu. AHBA microarray transcriptomic data are available at http://human.brain-map.org/static/download.

## Ethics Statement

Ethical review and approval was not required for the study on human participants in accordance with the local legislation and institutional requirements. Written informed consent for participation was not required for this study in accordance with the national legislation and the institutional requirements.

## Author Contributions

RM and DK formulated the hypothesis, designed the study, and wrote the manuscript. RM performed the bioinformatics analysis. Both authors contributed to the article and approved the submitted version.

## Conflict of Interest

The authors declare that the research was conducted in the absence of any commercial or financial relationships that could be construed as a potential conflict of interest.

## Publisher’s Note

All claims expressed in this article are solely those of the authors and do not necessarily represent those of their affiliated organizations, or those of the publisher, the editors and the reviewers. Any product that may be evaluated in this article, or claim that may be made by its manufacturer, is not guaranteed or endorsed by the publisher.

## Footnotes

1
http://ida.loni.usc.edu

2
https://github.com/chrisgorgo/alleninf
